# Establishment of a rat ovarian peritoneal metastasis model to study pressurized intraperitoneal aerosol chemotherapy (PIPAC)

**DOI:** 10.1186/s12885-019-5658-5

**Published:** 2019-05-07

**Authors:** Leen Van de Sande, Wouter Willaert, Sarah Cosyns, Kaat De Clercq, Molood Shariati, Katrien Remaut, Wim Ceelen

**Affiliations:** 1 Laboratory of Experimental Surgery, Department of Human Structure and Repair, Ghent University, Ghent, Belgium; 2 Cancer Research Institute Ghent (CRIG), Ghent University, Ghent, Belgium; 3 Laboratory of Pharmaceutical Technology, Department of Pharmaceutics, Ghent University, Ghent, Belgium; 4 Laboratory for General Biochemistry and Physical Pharmacy, Department of Pharmaceutics, Ghent University, Ghent, Belgium; 5 Department of GI Surgery, Ghent University Hospital, route 1275, C. Heymanslaan 10, B-9000 Ghent, Belgium

**Keywords:** PIPAC, ePIPAC, Ovarian cancer, Peritoneal metastases, Laparoscopic surgery, Intraperitoneal drug delivery, Rat xenograft

## Abstract

**Background:**

pressurized intraperitoneal aerosol chemotherapy (PIPAC), with or without electrostatic precipitation (ePIPAC), was recently introduced in the treatment of peritoneal metastases (PM) from ovarian cancer (OC). Preliminary clinical data are promising, but several methodological issues as well the anticancer efficacy of PIPAC remain unaddressed. Here, we propose a rat ePIPAC model that allows to study these issues in a clinically relevant, reproducible, and high throughput model.

**Methods:**

laparoscopy and PIPAC were established in healthy Wistar rats. Aerosol properties were measured using laser diffraction spectrometry based granulometric analyses. Electrostatic precipitation was accomplished using a commercially available generator (Ultravision™). A xenograft model of ovarian PM was created in athymic rats using intraperitoneal (IP) injection of SKOV-3 luciferase positive cells. Tumor growth was monitored weekly by in vivo bioluminescence imaging.

**Results:**

PIPAC and electrostatic precipitation were well tolerated using a capnoperitoneum of 8 mmHg. All rats survived the (e)PIPAC procedure and no gas or aerosol leakage was observed over the entire procedure. With an injection pressure of 20 bar, granulometry showed a mean droplet diameter (D(v,0.5)) of 47 μm with a flow rate of 0.5 mL/s, and a significantly lower diameter (30 μm) when a flow rate of 0.8 mL/s was used. Experiments using IP injection of SKOV-3 luciferase positive cells showed that after IP injection of 20 × 10^6^ cells, miliary PM was observed in all animals**.** PIPAC was feasible and well supported in these tumor bearing animals.

**Conclusions:**

we propose a reproducible and efficient rodent model to study PIPAC and ePIPAC in OC xenografts with widespread PM. This model allows to characterize and optimize pharmacokinetic and biophysical parameters, and to evaluate the anti-cancer efficacy of (e)PIPAC treatment.

## Background

Selected patients with peritoneal metastases (PM) benefit from cytoreductive surgery (CRS) combined with hyperthermic intraperitoneal chemoperfusion (HIPEC) [1, 2]. However, many patients present with irresectable disease, which has a dismal prognosis. Survival in patients with irresectable PM from colon cancer is 15 months, from gastric cancer 4 months, and from pancreatic cancer only 6 weeks [3–5]. Systemic chemotherapy is relatively inefficient in PM due to poor vascularity of peritoneal tumor nodules [6, 7]. Pressurized intraperitoneal aerosol chemotherapy (PIPAC) is a novel locoregional treatment modality which involves intraperitoneal (IP) delivery of chemotherapy as an aerosol during laparoscopic surgery [7, 8]. Chemotherapy is aerosolized in the abdominal cavity using a high-pressure injector and a nebulizer (Capnopen®). This method allows aerosolized chemotherapy to interact directly with tumor tissue. In addition, the elevated intra-abdominal pressure may enhance tumor tissue drug penetration [9].

Despite the significant potential of PIPAC and the preliminary clinical data already available, many technological and anti-cancer properties of the technique remain to be elucidated. In recent clinical practice, PIPAC was combined with electrostatic aerosol precipitation using the Ultravision™ system [10]. This device, originally developed to clear smoke from the laparoscopic operating field by an electrostatic force, uses a stainless-steel microfilament brush (Ionwand™) which is inserted into the abdominal cavity [11]. A high DC voltage (7.5–9.5 kV, ≤ 10 μA) is applied to the Ionwand, resulting in a corona discharge and a stream of negatively charged ions, which attach to suspended particles. These now negatively charged aerosol particles are attracted to the positive charge of the return electrode. In theory, the combination of electrostatic precipitation with PIPAC, termed ePIPAC, can result in increased tissue uptake of the aerosolized chemotherapy. However, this theoretical advantage remains to be confirmed in well-designed preclinical as well as clinical studies [12].

Several in vitro experimental PIPAC models have been described. The cytotoxic efficacy of PIPAC was investigated in vitro using proliferation assays of human colon cancer cells [13]. An in vitro model consisting of aerosol generation in a plastic box that contains human peritoneum with PM was used to study tissue penetration of doxorubicin, as well as the effect of treatment parameters such as nebulizer position, pressure, and drug dose [14, 15]. Schnelle et al. developed an interesting ex vivo model that consists of an inverted bovine urinary bladder, resulting in a serosa-lined cavity that can be used to study PIPAC [16]. Solass and coworkers demonstrated the technical proof of principle of PIPAC in a pig model [17]. However, the pig model is cumbersome, labour intensive, and expensive, and does not allow to study anticancer properties in xenografted or syngeneic PM. Here, we report the first small animal model of PIPAC and ePIPAC that allows to study several important endpoints such as tissue penetration and anti-cancer efficacy in PM of human origin.

## Methods

### Granulometric analyses

Volume-weighted particle size distribution (PSD) of aerosol droplets was performed in triplicate by laser diffraction (Mastersizer S long bench, Malvern Instruments, Worcestershire, United Kingdom). The size of aerosol droplets was measured in an open laser beam (water vs. air, refractive index of 1.33 and 1.00 respectively) using a 300F lens (0.5–900 μm) over a time horizon of 10 to 20 s after initiation of injection. Saline was nebulized using a commercially available nebulizer (Capnopen®, Capnomed, Zimmern, Germany) and a high-pressure injector (Injektron™ 82 M, Medtron, Saarbrücken, Germany). The outlet of the nebulizer was perpendicularly secured at a distance of 35 mm to the beam and 100 mm to the lens. The laser diffraction measurements were performed as soon as the high-pressure injector achieved the desired injection conditions, i.e. 10 s after initiating the injection. Results are expressed by median of volume distribution, D(v,0.5), i.e. the size at which 50 vol% of the droplets were either finer or coarser than the predicted value, with standard deviation.

### Ex vivo simulation of PIPAC

A 12 mm balloon trocar **(**Kii, advanced fixation sleeve, Applied Medical, Amersfoort, The Netherlands) was inserted in a closed 100 mL ethylene vinyl acetate (EVA) bag. CO_2_ was insufflated to establish a constant pressure of 8 mmHg. Twenty mL of undiluted royal blue ink (Pelikan nv, Groot-Bijgaarden, Belgium) was nebulized into the EVA bag with a flow rate of 0.8 mL/s and a maximal upstream injection pressure of 20 bar. The nebulizer was fixed either perpendicularly to the surface of the EVA bag, or in a slightly tilted position.

### Animals

Adult Wistar Hannover rats (*n* = 3 preliminary experiment; *n* = 6 in vivo experiment; Envigo, Horst, The Netherlands) and adult athymic nude rats (*n* = 9; Envigo, Horst, The Netherlands) were allowed to acclimatize to the surroundings for at least three days and were kept in standard housing conditions with water and food ad libitum and a 12 h light/dark cycle. After the experiments, all rats were euthanized with a lethal injection of T-61 (0.3 mL/kg, IV) into the tail vein. The experiments were approved by the Animal Ethical Committee of the Faculty of Medicine of Ghent University, Belgium (ECD 17–50 and ECD 18–30).

### Surgical methods

#### Experimental protocol of PIPAC in rats

The experimental procedure of PIPAC in the rat was based on clinical treatment protocols [18, 19]**.** Adult Wistar Hannover rats (*n* = 6) were anesthetized with sevoflurane (8 vol% induction, 4 vol% maintenance). Animals were placed in a class II laminar flow hood (Airstream, Esco Global, Barnsley, United Kingdom) in a supine position and fixed at all four extremities. The abdomen was shaved and disinfected. Next, a 5 mm and a 12 mm balloon trocar were inserted and a constant capnoperitoneum pressure of 8 mmHg was established (Olympus UHI-3 insufflator, Olympus Surgical Technologies Europe, Hamburg, Germany). Each trocar was secured by a tripod. The nebulizer was then connected to the high-pressure injector through a high-pressure line and inserted into the abdominal cavity using the 12 mm trocar. Afterwards, a 5 mm laparoscope was introduced into the abdominal cavity using the 5 mm trocar. The tightness of the abdomen was documented via absence of CO_2_ flow. Next, the high-pressure injector was activated. Injection parameters were set at a flow rate of 0.8 mL/s and a maximal upstream injection pressure of 20 bar. When used with the nebulizer, the high-pressure injector needs up to 10 s to achieve the desired conditions (flow rate and injection pressure). Therefore, the high-pressure injector was set to inject 8 mL of air during these first 10 s, before nebulizing 20 mL of saline at the desired conditions. The capnoperitoneum pressure of 8 mmHg was maintained for 30 min. Thereafter, the aerosol was evacuated through a closed aerosol waste system containing a 99.999% ULPA-carbon filter. Finally, trocars were removed, and the laparoscopic procedure was terminated. The abdomen was closed with a two-layered running suture (Vicryl Plus 4–0 Ethicon, Johnson & Johnson international, Sint-Stevens-Woluwe, Belgium) and analgesia was administered (Ketoprofen, 5 mg/kg, SC).

#### Electrostatic precipitation

**Fig. 1:**
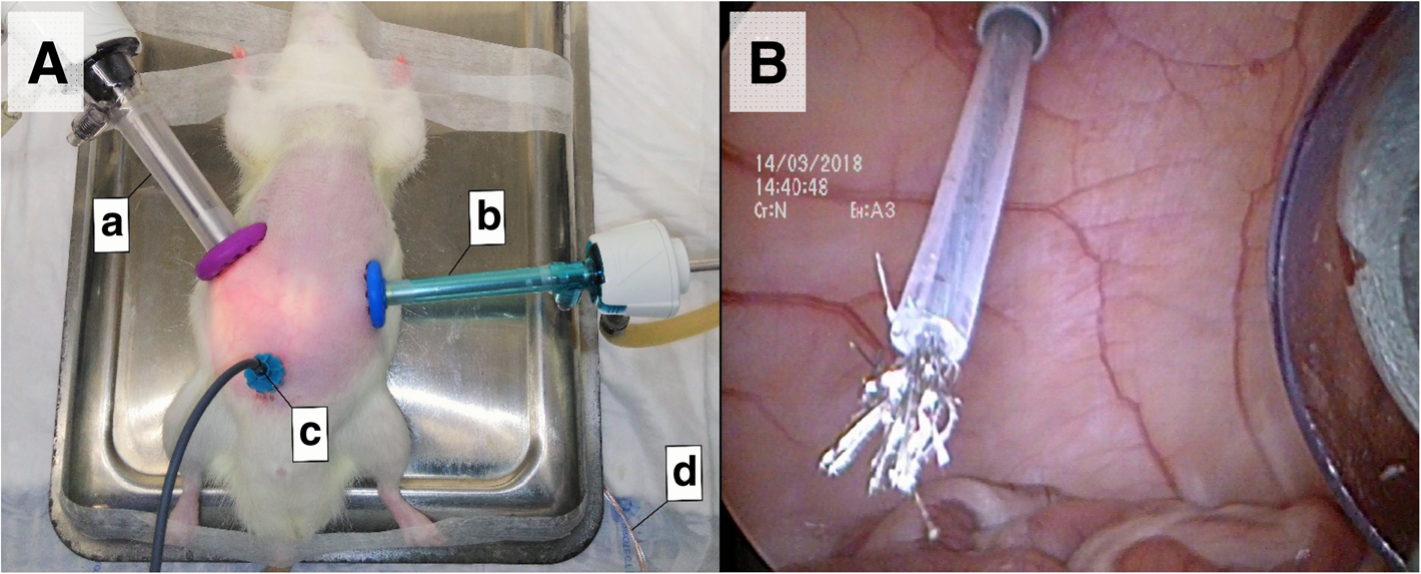
**A** ePIPAC setup in a healthy Wistar Hannover rat. 12 mm balloon trocar with nebulizer and closed aerosol waste system (a), 5 mm balloon trocar with laparoscope and CO_2_ insufflator (b), Ionwand (c), electrical conductor between return electrode (underneath metal plate) and generator unit (d). **B** Intra-abdominal view of the Ionwand in a healthy Wistar Hannover rat

In three adult Wistar Hannover rats, the use of Ultravision™ technology (Alesi Surgical, Cardiff, United Kingdom) was added to the PIPAC-setup. The system was activated at the start of aerosol generation and the electric current was maintained for 30 min. The Ultravision™ system integrates the following components: a generator unit (voltage 7500–9500 V, current ≤ 10 μA), an active cable terminating in an atraumatic stainless-steel brush electrode (Ionwand) that is responsible for the electrostatic charging of aerosol particles, and a return electrode with a solid return plate. To obtain a closed electrical circuit, the solid return plate was fixed under a metal plate. During the ePIPAC procedure, the rat was positioned on this metal plate (Fig**.** 1).

### Rat ovarian PM model

SKOV-3 Luc IP2 cells, a human ovarian carcinoma cell line created by double in vivo selection of SKOV-3 Luc cells, were used to create a rat xenograft model [20]. The cells (kindly donated by Olivier De Wever, Laboratory of Experimental Cancer Research, Ghent University) were cultured at 37 °C in a 10% CO_2_ containing atmosphere in McCoy’s 5A medium (ThermoFisher Scientific Bvba, Merelbeke, Belgium) supplemented with 10% fetal bovine serum, penicillin and streptomycin (ThermoFisher Scientific Bvba, Merelbeke, Belgium).

Athymic nude rats (*n* = 9) were injected IP with 5 × 10^6^ (*n* = 3), 10 × 10^6^ (n = 3) or 20 × 10^6^ (n = 3) SKOV-3 Luc IP2 cells in a volume of 5 mL saline. The rats underwent a daily subcutaneous injection of cyclosporine (3 mg) starting three days prior to, and until four weeks after tumor cell inoculation to ensure tumor growth. Tumor growth was monitored by weekly in vivo bioluminescent imaging (BLI) (IVIS Lumina II, PerkinElmer, Zaventem, Belgium). Imaging was performed 12 min after IP injection of D-luciferin (120 mg/kg; PerkinElmer, Zaventem, Belgium). The exposure time was one second and the binning (resolution setting) was set to medium.

After four weeks, PIPAC and ePIPAC with saline were performed according to the abovementioned experimental procedures. The rats were euthanized 24 h after (e)PIPAC with a lethal injection of T-61 (0.3 mL/kg, IV) into the tail vein**.** Peritoneal tumors were excised and embedded in paraffin for standard hematoxylin and eosin (H&E) staining.

### Statistical analysis

Differences between D(v,0.5) values were compared using the non-parametric Mann-Whitney U test since data were not normally distributed. A *p*-value of ≤ 0.05 was considered to indicate statistical significance.

## Results

### Influence of physical parameters on aerosol formation

**Fig. 2:**
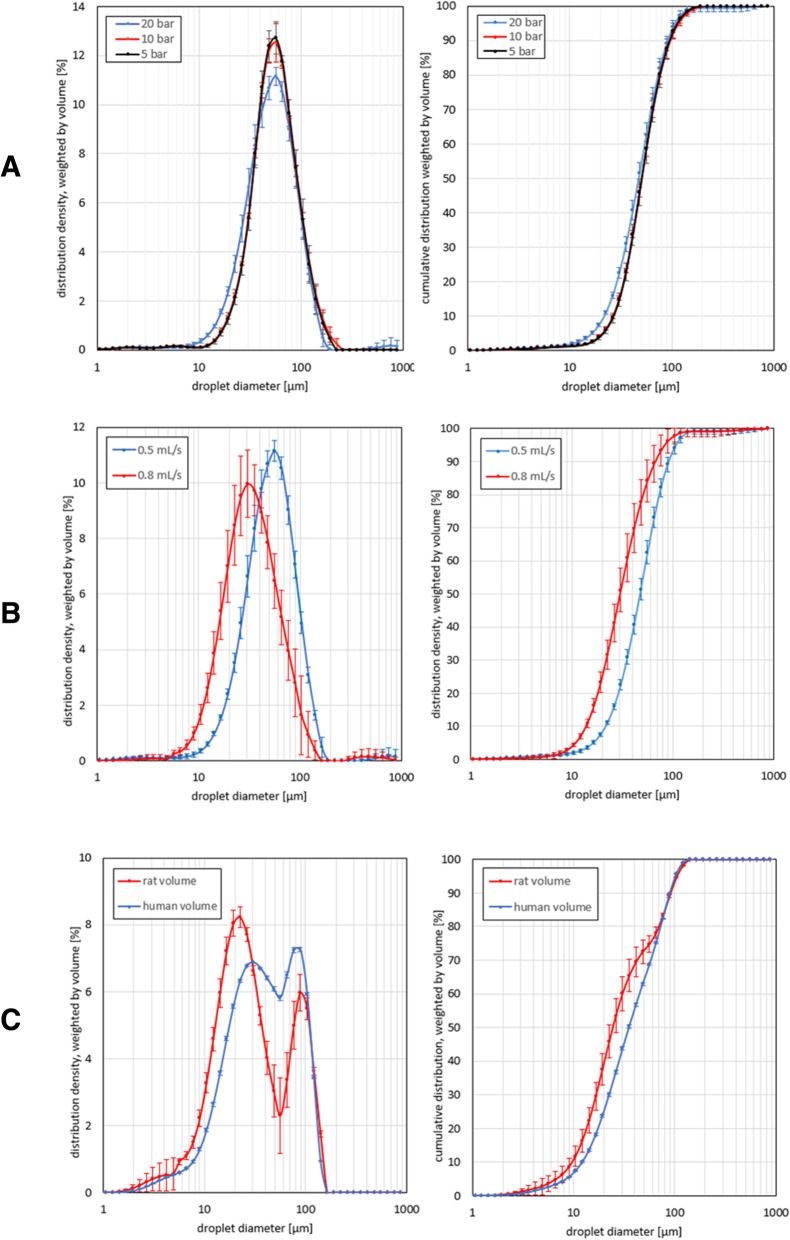
PSD curves of nebulized saline showing the distribution density (left curves) and cumulative distribution (right curves). Mean droplet diameters were measured (*n* = 3 for each confirmation) in a range of 0.5 to 900 μm. The error bars show one time the standard deviation. **a** Volume-weighted PSD curves showing the influence of maximal upstream injection pressure on D(v,0.5). 20 mL of saline was nebulized in open space at a fixed flow rate of 0.5 mL/s and a maximal upstream injection pressure of 20 bar (blue graph), 10 bar (red graph) or 5 bar (black graph). **b** Volume-weighted PSD curves illustrating the influence of flow rate on D(v,0.5). 20 mL of saline was nebulized in open space at a fixed maximal upstream injection pressure of 20 bar and a flow rate of 0.5 mL/s (blue graph) or 0.8 mL/s (red graph). **c** Volume-weighted PSD curves demonstrating the influence of the volume of the peritoneal cavity on D(v,0.5). To simulate a nebulization in a rat’s abdominal cavity, 20 mL of saline was nebulized in a plastic box (V = 100 mL) with a flow rate of 0.8 mL/s and a maximal upstream injection pressure of 20 bar (red graph). 200 mL of saline was nebulized in a plastic box (V = 5 L) with a flow rate of 0.5 mL/s and a maximal upstream injection pressure of 20 bar (blue graph) as a comparison with the nebulization in human. The plastic boxes were bilaterally pierced to transvers laser light and a third perforation was made at the top of the boxes to insert the nebulizer

A maximal upstream injection pressure of 20 bar and a fixed flow rate of 0.5 mL/s were associated with a D(v,0.5) of 47 ± 2 μm (Fig. 2a). Decreasing the maximal upstream injection pressure to 10 bar, resulted in an increased D(v,0.5) of 51 ± 1 μm (*p* = 0.127). However, when the maximal upstream injection pressure was further decreased to 5 bar, D(v,0.5) remained 51 ± 2 μm (*p* = 0.127).

Analysis of different flow rates (0.5 and 0.8 mL/s) at a fixed maximal upstream injection pressure of 20 bar, showed a strong impact on the volume-weighted PSD curves (Fig. 2b). Applying a flow rate of 0.5 mL/s, D(v,0.5) was 47 ± 2 μm. Increasing the flow rate to 0.8 mL/s resulted in a decreased D(v,0.5) of 30 ± 3 μm (*p* = 0.05).

Bimodal volume-weighted PSD curves were observed when nebulization of saline was done in a closed environment using a plastic box (Fig. 2c). Aerosol formation in patient-like conditions, i.e. nebulization of 200 mL saline with a maximal upstream injection pressure of 20 bar and a flow rate of 0.5 mL/s in a plastic box of 5 L, showed a D(v,0.5) of 35 ± 1 μm. After nebulization with rat-like conditions, i.e. nebulization of 20 mL with a maximal upstream injection pressure of 20 bar and a flow rate of 0.8 mL/s in a plastic box of 100 mL, a D(v,0.5) of 25 ± 3 μm (*p* = 0.05) was detected.

**Fig. 3:**
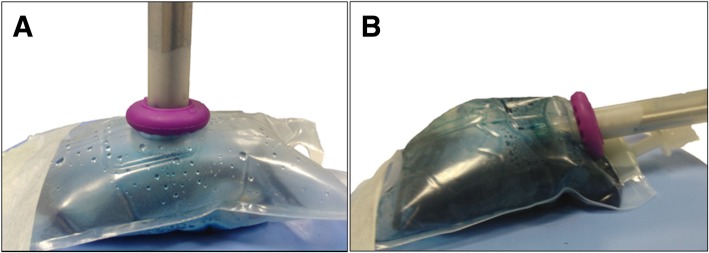
Distribution pattern of 20 mL undiluted royal blue ink. The injection parameters were set on a flow rate of 0.8 mL/s, a maximal upstream injection pressure of 20 bar and an intracavitary pressure of 8 mmHg. **a** Blue ink distribution was limited to the bottom of the EVA bag when the nebulizer was perpendicularly secured. **b** Complete staining of the EVA bag was observed when the nebulizer was placed in a tilted position

Adequacy of stain distribution throughout the entire EVA bag was clearly superior with a slightly tilted nebulizer position (Fig. 3). Specifically, the top of the EVA bag was stained when the nebulizer was secured in a tilted position, while this was not the case when it was placed perpendicularly.

### Experimental PIPAC procedure

Once the ideal injection parameters for (e)PIPAC were determined, PIPAC with saline was tested in healthy Wistar Hannover rats (*n* = 3). The feasibility of repeated PIPAC applications was successfully tested in these preliminary experiments. The three rats underwent three consecutive PIPAC procedures with an interval of four weeks. The rats tolerated multiple PIPAC applications very well and were in good health after the third PIPAC application. Thereafter, PIPAC (n = 3) and ePIPAC (n = 3) was performed in healthy adult Wistar Hannover rats. Procedures were executed as planned in both groups. The nebulizer enabled rapid and effective nebulization of saline in the abdominal cavity without extracorporeal leakage of aerosolized droplets over the entire setup. All rats survived the procedures.

### Rat ovarian PM model

**Fig. 4:**
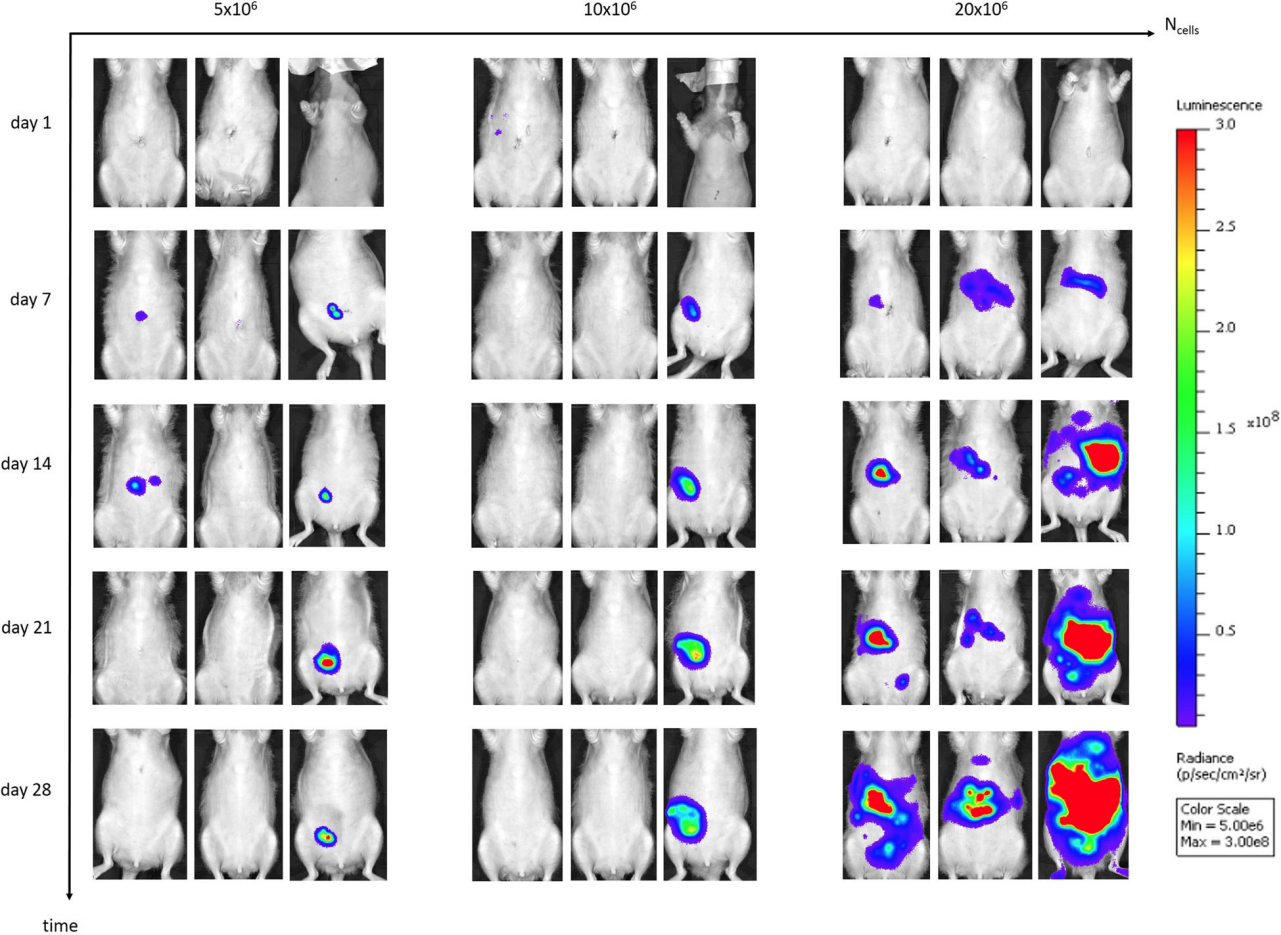
In vivo BLI of athymic nude rats inoculated with 5 × 10^6^ (group 1), 10 × 10^6^ (group 2) or 20 × 10^6^ (group 3) SKOV-3 Luc IP2 cells (n = 3 in each group). In the first and second group no diffuse PM was observed. One rat demonstrated tumor clearance, two rats had only one tumor nodule and in the other specimens no tumor growth occurred. In the third group diffuse PM was observed with a tumor induction rate of 100%

In vivo BLI of tumor-bearing rats is illustrated in Fig. 4. In the **first (**5 × 10^6^ SKOV-3 Luc IP2 cells) and second group (10 × 10^6^ SKOV-3 Luc IP2 cells), no diffuse PM was observed. After four weeks, two rats had only one tumor nodule and in the other specimens no tumor growth occurred. However, in the third group (20 × 10^6^ SKOV-3 Luc IP2 cells) miliary PM was observed with a tumor induction rate of 100% (3/3).

**Fig. 5:**
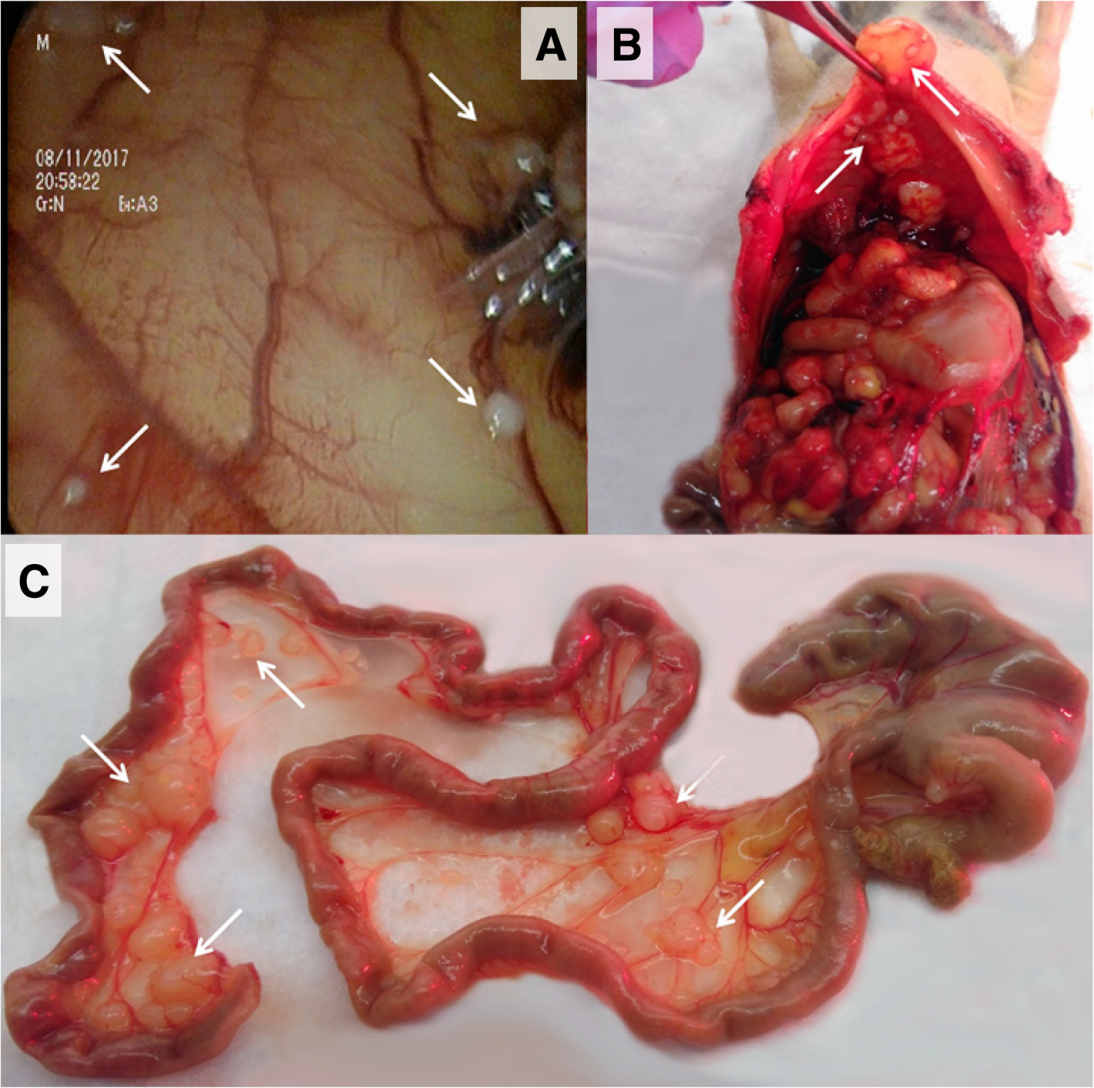
Tumor nodules (white arrows) in athymic nude rats inoculated intraperitoneally with 20 × 10^6^ SKOV-3 Luc IP2 cells. **a** Laparoscopic image of the right upper abdomen. **b** Post-mortem view of the upper abdomen. **c** Intestines and mesentery

**Fig. 6:**
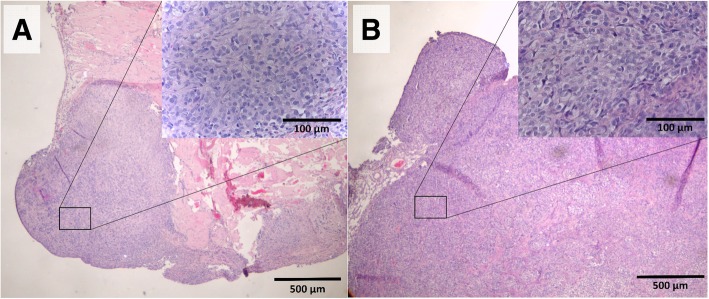
H&E stainings of peritoneal implants at the parietal peritoneum (**a**) and mesentery (**b**), 40x magnification. In the right upper corner, a close up of the H&E staining is shown, 400x magnification. The typical morphology of cancer cells can be detected

To assess whether it was possible to perform (e)PIPAC in tumor bearing rats, this procedure was performed with saline on day 28 in the athymic nude rats of group 3. All athymic nude rats survived this procedure. On day 29, the rats of group 3 were sacrificed and autopsy was done. Diffuse PM with widespread small tumor nodules could be demonstrated (Fig. 5). Tumor nodules were embedded in paraffin before standard H&E staining was conducted. H&E staining confirmed the presence of tumor cells in the nodules (Fig. 6).

## Discussion

The prognosis of patients with irresectable PM is poor, with a median survival ranging from several weeks to months [3–5]. Recently, PIPAC has been introduced in clinical practice [21]. During PIPAC, chemotherapy is delivered as an aerosol, generated by a nebulizer (Capnopen®) connected to a high-pressure injector. This novel approach may overcome two major limitations of conventional liquid based IP chemotherapy: incomplete coverage the peritoneal surfaces and poor tissue drug penetration [22, 23]. The increased intra-abdominal pressure during PIPAC by means of the capnoperitoneum may overcome the high interstitial fluid pressure in tumor tissue, and therefore improve tissue penetration [9]. Also, IP delivery of therapeutic agents as an aerosol result in a more homogeneous distribution compared to liquid instillation [12, 17, 21]. However, the biophysical, pharmacokinetic, and anticancer properties and parameters that are relevant in PIPAC remain relatively unknown. To date, four models were reported for (e)PIPAC research: in vitro using cancer cells, an ex vivo box model, an ex vivo bovine urinary bladder model and an in vivo pig model [13–17, 24, 25]. We report a standardized rat model that allows to study biophysical, pharmacokinetic, and anticancer properties of PIPAC and electrostatic precipitation combined with PIPAC.

The first step was to ascertain that the model generates an aerosol that is comparable to the clinical situation. In clinical practice, the recommended settings are: nebulization of a volume of 150 to 200 mL, maximal upstream injection pressure of 20 bar, flow rate of 0.5 mL/s and a capnoperitoneum pressure of 12 mmHg [21]. The resulting aerosol droplet size is reported to be in the range of 30–40 μm [26]. In vitro granulometric analyses performed by Göhler et al. showed that the Capnopen® generated aerosol consists of a bimodal volume-weighted PSD with a D(v,0.5) of 25 μm [27]. The authors argue, based on theoretical assumptions and calculations, that the ideal droplet size to obtain a gas-like behaviour should be 1.2 μm (for Stokes numbers of Stk = 1) [27]. The droplet size of the aerosol is indeed important for its physical behaviour in the peritoneal cavity (diffusion versus sedimentation or inertial impaction), but may also affect tissue penetration of the administered cytotoxic agent [27, 28]. In pulmonary medicine, only very small (< 5 μm) particles reach the alveoles and exert a meaningful therapeutic effect. [29–31] It should be noted that smaller particles carry less drug mass, and that the anatomical restrictions that apply to the respiratory tree do not apply to the peritoneal cavity. For instance, large (> 10 μm) inhaled particles tend to be deposited in the larynx or upper airways, which is undesirable, but in PIPAC treatment size considerations seem to be less important. Nevertheless, the current and ideal PIPAC droplet size, and the biophysical parameters that affect it, should be further characterized. In our model, analysis of volume-weighted PSD curves demonstrated that D(v,0.5) is 47 μm when a flow rate of 0.5 mL/s and a maximal upstream injection pressure of 20 bar are used (Fig. 2a). Increasing the flow rate to 0.8 mL/s resulted in a statistically significant decrease of D(v,0.5) to 30 μm, an observation in accordance with experience from nozzles used in inlet fogging of gas turbine engines [32]. Interestingly, after nebulization of saline in a plastic box, a bimodal volume-weighted PSD distribution curves was observed, possibly explained by aggregation of the injected aerosol droplets.

Next, we studied homogeneity of aerosol distribution using a dye. It was observed that distribution of undiluted royal blue ink is improved when the nebulizer was held in a slightly tilted position; this could be explained by a longer travelling distance of the injected aerosol droplets compared to the perpendicular position.

For the in vivo experiments, the pneumoperitoneum pressure was set at 8 mm of Hg, since it is well known that an intra-abdominal pressure of 12 mmHg is poorly tolerated in rats [33, 34]. Preliminary results showed excellent tolerance of both PIPAC and electrostatic precipitation. With experience, the total procedure time (from start to end of anesthesia) was ±100 min. The ovarian cancer xenograft model, resulting in widespread military PM, was successfully established after increasing the number of SKOV-3 Luc IP2 cells to 20 × 10^6^ and with concurrent administration of cyclosporin. The required number of cells may appear high, but it is known that athymic rats have a lower degree of immune deficiency compared to athymic mice [35]. This model now offers the opportunity to study the anticancer effects of (e)PIPAC either alone or in combination with systemic treatment. Since immunotherapy offers considerable promise in ovarian cancer, the availability of a syngeneic OC model in immune competent animals would allow to study PIPAC in combination with immunotherapy. A syngeneic rat OC model, based on IP injection of NuTu-19 cells in female Fischer 344 rats, has been described [36], and may represent an interesting future addition to the xenograft model that we propose. Also, the use of immune competent animals allows to study how PIPAC treatment affects the peritoneal immune environment, a relevant parameter in the biology of post-therapy recurrence.

The major limitation of our model is related to the large difference in size between the rat and the human peritoneal cavity. This requires scaling of the effects found, and some clinical apparatus and methods may work differently when used in a rat. As an example, when using the clinically used electrostatic generator for ePIPAC in a rat, the shorter distance between electrode and grounding plate will result in a much stronger electrical field (volt/meter). In the future, we will study ePIPAC using a custom made apparatus that allows to vary voltage, current, and polarity.

## Conclusion

We report the first reproducible small animal model of PIPAC and ePIPAC in human ovarian PM bearing rats. This model will allow to study essential technology related aspects, pharmacokinetics and tissue penetration, and the activity of aerosolized anticancer therapies.

## Abbreviations

BLI: Bioluminiscent imaging
CRS: Cytoreductive surgery
D(v,0.5): Median of volume distribution
ePIPAC: Electrostatic aerosol precipitation with PIPAC
EVA: Ethylene vinyl acetate
H&E: Hematoxylin and eosin
HIPEC: Hyperthermic intraperitoneal chemoperfusion
IP: Intraperitoneal
OC: Ovarian cancer
PIPAC: Pressurized intraperitoneal aerosol chemotherapy
PM: Peritoneal metastases
PSD: Particle size distribution
Stk: Stokes number
